# The fertility effects of public pension: Evidence from the new rural pension scheme in China

**DOI:** 10.1371/journal.pone.0234657

**Published:** 2020-06-12

**Authors:** Zheng Shen, Xiaodong Zheng, Hualei Yang

**Affiliations:** 1 School of Economics and Management, Zhejiang A&F University, Hangzhou, China; 2 Key Cultivating Think Tank of Zhejiang Province—Research Academy for Rural Revitalization of Zhejiang Province, Zhejiang A&F University, Hangzhou, China; 3 School of Economics, Zhejiang Gongshang University, Hangzhou, China; 4 School of Public Administration, Zhongnan University of Economics & Law, Wuhan, China; Xiamen University, CHINA

## Abstract

Public pension insurance has become a major form of social protection around the world. However, little is known about the association between public pension expansion and individuals’ fertility in developing economies. In this paper, we examine the effects of the New Rural Pension Scheme (NRPS) on the fertility of married women in rural China. Using data from the China Family Panel Studies (CFPS), the difference-in-differences approach is employed to estimate the impact of NRPS expansion on fertility outcomes. The robustness of results is checked through additional estimations, including difference-in-differences with propensity score matching, fixed-effects model, and instrumental variable approach. Results show that the NRPS expansion has a significantly negative effect on the number of children, and it reduces the likelihood of having a second child. The fertility-reducing effect of the NRPS is larger for the younger, well-educated women and those in high-income families. Considerations of the fertility effects and their population differences are needed in the impact evaluations of relevant public pension reform.

## Introduction

Public pension insurance is considered as one of the major social security programs to improve social welfare and reduce inequalities. The expansion in pension coverage of young adults affects the lifetime budget constraint. This, in turn, may lead to changes in individual behavior, such as fertility. While numerous studies of the association between the old-age pension and fertility have been conducted for industrialized countries [1–6] the fertility effects of public pension for rural women in developing regions have received less attention.

China is the world’s largest developing economy and has achieved rapid industrial and economic growth during the past few decades. According to the World Bank Open Data (WBOD), however, it has seen the percentage of population aged 65 and above increase sharply over the last years, from about 5.63% in 1990 to 10.35% in 2017. Meanwhile, the total fertility rate decreased from 2.3 in 1990 to 1.6 in 2015, which is lower than the replacement-level fertility. The shifts of population structure in China reflect a demographic transition from a population pattern previously characterized by demographic dividend and large working-age population to low fertility rates and aging population, raising considerable concerns about the pension arrangement and sustainability. Yet, little is known about the Chinese people’s fertility responses to their country’s public pension system.

Researches in economics have assumed that the parents are altruistic toward their children, which are considered as a consumption good to produce utility for their parents [7–8]. Based on this assumption, Becker and Barro (1988) concluded a negative relationship between fertility and the growth rate of social security [7]. On the other hand, the egoism argued that parents view children as the investment good because of old-age security motive for fertility [1]. Nonetheless, pension benefits represent a substitute for the intergenerational transfer from children and thus reduce parental childbearing intentions [9]. In a neoclassical growth model, Miyazaki (2013) theoretically analyzed how unfunded social security influences economic growth, fertility rate, and welfare [10]. The results showed that the impact of this social security on fertility depends not only on the size of the monetary cost relative to the time spent on child-rearing, but also on the current fertility and interest rate in laissez-faire. By extending the overlapping generations (OLG) model, Wang (2015) revealed that, for any given minimum wage, the public pension system may improve fertility and decrease unemployment [11].

Several studies have empirically examined the impacts of social security or public pension on fertility, and most of them revealed a negative association. For instance, Cigno (1992, 1993) found that the expansion of social security coverage has a significant negative impact on fertility in European countries [1][12]. Using data from 49 countries over 29 years, Ehrlich and Zhong (1998) found that social security tax and expenditure on social security projects tend to crowd out the household fertility [2]. Boldrin et al. (2015) obtained a similar result in the U.S. and Europe, where there was a strong negative correlation between the size of social security and the total fertility rate [3]. Ehrlich and Kim (2007) used panel data from 57 countries over 32 years, and demonstrated that the pay-as-you-go (PAYG) pension system accounted for a sizable part of the worldwide downward trend in fertility and family formation, as well as a slowdown in saving rates and economic growth [4]. The findings of Fenge and Schubel (2016) also confirmed that a PAYG pension is negatively associated with the fertility level based on historical data analyses of Imperial Germany [5].

Since the public pension system plays an important role in protecting against the future earnings uncertainty, young adults may adjust their saving for old-age consumption to maximize utility throughout their lifetime [13][14]. On the other hand, the life-cycle model predicts that an increase in future pension wealth would be offset by a decline in savings. With regard to China, Feng et al. (2011) used the exogenous variation in pension wealth to estimate the impact of pension wealth on household savings, and found a significant offset effect of pension wealth on household savings [15]. Meanwhile, results from studies of Lugauer et al. (2019) shown that Chinese families with fewer children have significantly higher saving rates [16]. Thus, given that the pension’s effect on fertility might be crowded out when the pension wealth is offset by the change of savings, the role of saving decisions in the association between public pension and individuals’ fertility needs to be considered.

This paper attempts to fill the gap of existing research concerning the link between public pension and fertility. First, different from the previous studies, we offer a new insight into the fertility preferences among the rural women of a rapidly growing country, China. This study extends existing analysis on the fertility effect of pension, which has largely focused on advanced countries. Second, we use individual-level microdata from a nationally representative survey to empirically test the hypotheses derived from the theoretical model, revealing the fertility differences between pension and non-pension scenarios. We also employ a series of identification strategies to examine the causal effect of public pension insurance on individuals’ fertility, and investigate whether the effect varies by population groups.

Since 2009, the Chinese government has expanded public pension availability for rural residents. Using data from 2010 and 2014 waves of the China Family Panel Studies, we employ a difference-in-differences strategy to examine the fertility effects of New Rural Pension Scheme on targeted populations. The actual number of children and whether to have a second child are the two indicators used to measure the fertility outcomes among rural married women aged between 20 and 45. Moreover, we implement difference-in-differences with propensity score matching, fixed-effects model, and instrumental variable approach to check the validity of our results and analyze the heterogeneity of treatment effects by age, education, and household income. Our empirical findings, which are robust to several specification checks, indicate that the implementation of rural pension scheme has a significantly negative effect on the number of children, and it reduces the likelihood of having a second child. The fertility-reducing effect of the public pension is larger for the younger, well-educated women and those in high-income families. By contrast, the rural pension expansion does not significantly reduce women’s fertility for the elderly, people with less education, and low-income families.

The remainder of this paper is organized as follows. Section 2 describes the institutional background; Section 3 provides a conceptual framework. Section 4 presents the data source and the main empirical strategy. Section 5 describes the main results including descriptive statistics, the effect of the NRPS on fertility and subgroups analyses, robustness checks (sample selection and endogeneity). Lastly, discussion and concluding remarks are in Section 6.

## Institutional background

China’s public pension system is comprised of three main types of pension scheme: the Urban Employees’ Pension Plan (UEPP), the New Rural Pension Scheme (NRPS), and the Urban Resident Pension Scheme (URPS). Among them, the NRPS is designed for covering rural residents aged 16 years or above who are not students and not covered by the basic urban pension scheme. Before the NRPS, the Chinese government experimented with pilot programs to extend a rural pension scheme starting in 1986. The program placed most of the financial responsibility on individuals, was supplemented by local collectives, and covered both the collective and private sectors in rural China. However, this financial arrangement led to a very low take-up rate with a lack of sustainability and effectiveness of public financial support, and the pilot scheme expansion was halted in the late 1990s.

To improve the economic well-being of the rural elderly, the Chinese government launched the NRPS in 2009. Since its inception the NRPS has rapidly expanded, where participating counties have increased over time, from 838 in 2010 to nearly all 2,853 counties by 2012 and 326.4 million rural residents were participating in this scheme in 2011 [17]. Compared to the old rural pension system relying mainly on the collective commune, the NRPS is heavily subsidized by the central government and aiming to provide income protection for rural citizens (agricultural hukou holders) aged 60 or above who are not covered by other pension schemes [18]. It is voluntary for local rural citizens aged 16 or above to participate, and the participants must contribute to their account for 15 years to be eligible for a pension at age 60.

The pension returns of NRPS consist of the individual account benefits and a non-contributory basic pension. Individual account benefits are calculated based on the participant’s accumulated contributions from the individual account and accrued investment returns [19]. A minimum level of the basic pension is initially provided by the central government, and the amount will be further adjusted by local government according to their fiscal capacity. So the amount of the basic pension varies considerably between developed (as high as 370–380 CNY per month) and less-developed regions (only 55 CNY per month) [17]. Although the economic benefits of NRPS are relatively low compared to old-age pension programs in some other developing countries, it is not negligible that the NRPS could help rural family members maintain their basic living standards.

To control the growing population, the Chinese government introduced a birth planning program (the one-child policy) in 1979. The one-child rule was strictly enforced for urban residents. In rural areas, however, it was virtually unenforceable and from 1984 rural couples in most provinces were allowed to have a second child if their first was a girl. Particularly, rural couples from the six northwestern provinces were allowed a second child, irrespective of the sex of the first child [20]. In order to cope with the rapid population aging, since 2007 the one-child policy in China has been gradually relaxed, and all provinces (except Henan, which followed in 2011) allowed couples who were both only children themselves to have two children. In 2013, the policy was further adjusted for allowing married couples in which at least one of the partners was an only-child to have two children (the selective two-child policy). In October 2015, the Chinese government announced that a universal two-child policy was implemented to replace the country's previous one-child policy [20]. Overall, under the birth planning policy, women in rural China are allowed to have a second child as long as the couples meet the conditions of the two-child policy. In subsequent analyses, the association between rural pension expansion and the women’s second childbirth will be examined.

## Conceptual framework

We extend the overlapping generations (OLG) model, by incorporating public pension and saving, to investigate the fertility response of young adult to the change in pension coverage using comparative statics. In this model, the economy is populated by individuals who live through three periods: childhood, adult and old age. When the individual is a child, she lives with her parents who provide necessary inputs for child-rearing and development. In the period of old age, the individual is retired and her consumption consists of saving and pension income. Young adults, the main labor force and wage earner, acts as a sole role in making family decisions such as fertility and consumption.

Studies regarding fertility consider children relative to the presence of two attributes. First, children are to some extent a consumption good, which could produce utility for their parents [8]. Second, the egoism argued that parents view children as the investment good because of old-age security motive for fertility [1]. For this reason, we assume that each adult at generation *t* obtain utility (*U*_*t*_) from the number of children (*n*_*t*_) in the family as well as the consumption in young ($C_{t}^{1}$) and old age ($C_{t+1}^{2}$). Based on the works of Miyazaki (2013), the lifetime utility function of an individual of generation *t* is assumed to be additively separable and semi-logarithmic:

$$U_{t}=logC_{t}^{1}+\beta logC_{\left(t+1\right)}^{2}+\gamma logn_{t}$$ (1)

Here, *β* is a utility discount factor in a range between 0 and 1, while *γ*(*γ*>0) represents the utility weight of raising children relative to consumption. We first consider the scenario that the person does not participate in any pension scheme, and thus the individual in generation *t* has to face following the budget constraint:

$$C_{t}^{1}+S_{t}+pn_{t}+\phi w_{t}=w_{t}$$ (2)

Where *S*_*t*_ denotes the savings for consumption in the retirement period, *p* is the cost of each child's human capital inputs, *ϕ* (*ϕ*>0) is the rate of the expenditure to support the elderly, and *w*_*t*_ is an adult’s wage income. The budget constraint in the old age period is written as:

$$C_{t+1}^{2}=S_{t}R_{t+1}+n_{t}\phi w_{t+1}$$ (3)

Where *R*_*t*+1_ = 1+*r*_*t*+1_, and *r*_*t*+1_ is the interest rate in period *t*+1. Except for saving, consumption in old age also includes the pecuniary return to having children, represented by *n*_*t*_*ϕw*_*t*+1_. Maximizing one’s lifetime utility function as described in (1), while it subjects to budget constraints (2) and (3). We obtain the optimal solution of fertility level $n_{0}^{*}$, that is:

$$n_{0}^{*}=\frac{\gamma }{\left(1+\beta +\gamma \right)\left(pR_{t+1}-\phi w_{t+1}\right)}R_{t+1}\left(1-\phi \right)w_{t}$$ (4)

We next examine the fertility decisions for the individual who had pension coverage. Assuming that the contribution rate of social security involving the public pension is denoted by *θ*, then the budget constraint for young adults at generation *t* can be written as:

$$C_{t}^{1}+S_{t}+pn_{t}+\phi w_{t}=\left(1-\theta \right)w_{t}$$ (5)

Where (1−*θ*)*w*_*t*_ is an individual’s net wage, which is distributed into consumption, saving, money spent on child-rearing, and support for the elderly. In the retirement period, the individual’s consumption ($C_{t+1}^{2}$) come from return on young-period savings (*S*_*t*_*R*_*t*+1_), pension income from government (*G*), and pecuniary return to children (*n*_*t*_*ϕw*_*t*+1_). Hence, the budget constraint is:

$$C_{t+1}^{2}=S_{t}R_{t+1}+G+n_{t}\phi w_{t+1}$$ (6)

Maximizing the utility function (1) subjects to the budget constraints (5) and (6), the optimal fertility level $n_{1}^{*}$ under public pension scenarios is as follows:

$$n_{1}^{*}=\frac{\gamma }{\left(1+\beta +\gamma \right)\left(pR_{t+1}-\phi w_{t+1}\right)}\left[R_{t+1}\left(1-\phi \right)w_{t}+G-\theta R_{t+1}w_{t}\right]$$ (7)

In order to compare the fertility differences between pension and non-pension, we subtract the Eq (7) from Eq (4):

$$n_{1}^{*}-n_{0}^{*}=\frac{\gamma }{\left(1+\beta +\gamma \right)}\frac{\left(G-\theta R_{t+1}w_{t}\right)}{\left(pR_{t+1}-\phi w_{t+1}\right)}$$ (8)

To simplify the analysis, the contribution rate *θ* and the interest rate *R*_*t*+1_ are assumed to be constant. Since the parameters *p*,*β*,*γ*, and *R*_*t*+1_ are greater than zero, and 0<*θ*<1, the fertility response to pension plan depends on the sign of (*pR*_*t*+1_−*ϕw*_*t*+1_) and (*G*−*θR*_*t*+1_*w*_*t*_). The former shows the comparison between cost and pecuniary return of a child, while the latter reflects a tradeoff between future pension benefits and the current earnings. Particularly, if people are provided a high transfer income from public pension (i.e., *G*−*θR*_*t*+1_*w*_*t*_>0) and the economic return is more important for people to have children (i.e., *pR*_*t*+1_−*ϕw*_*t*+1_<0), having pension would generate large negative impacts on fertility. In the empirical analyses below, we will use survey data and employ identification strategies to examine the causal relationship between pension and fertility, and investigate whether the effect varies by population groups.

## Data and methods

### Data sources

The data used in the present study comes from the China Family Panel Studies (CFPS), a nationally representative and longitudinal survey conducted by the Institute of Social Science Survey (ISSS) of Peking University starting in 2010. A multi-sage probability proportional to size (PPS) strategy with implicit stratification was performed in the sampling process that comprises three stages: county level as the primary sampling unit, a community or village for the second-sage sampling unit and the final sampling unit was household [21]. The CFPS survey consists of a rich set of socio-economics questions and information on the levels of child, adult, family and community. The main variables used in our study are from adult and family questionnaires, which gather detailed information on demographic and family characteristics, employment status, social security, fertility history, as well as a series of pension-related questions such as types of pension schemes and the participation status. CFPS was undertaken according to the guidelines laid down in the Declaration of Helsinki and all procedures involving human participants were approved by the ethics committee of Peking University. Written informed consent was obtained from all participants.

Given that the NRPS coverage rapidly extended during 2011 and 2012, we construct a two-wave balanced panel data by merging the baseline in 2010 with a follow-up survey in 2014 from the adult and family dataset of CFPS, so as to compare the fertility outcomes of individuals with NRPS and non-NRPS before and after program implementation. To meet the requirements of participation in NRPS, the sample is restricted to tracked individuals having an agricultural Hukou, the eligibility for participation in the NRPS. Aside from NRPS, those who participated in another pension scheme, such as an urban and private pension, are excluded from the sample of our study. This is because other types of pension may also alter individual reproductive behaviors. Introducing these pension programs might cause a bias on the results for estimating the impact of NRPS on fertility. For the fertility analysis, we restricted the sample to married women aged between 20 and 45 because 20 is the legal minimum marriage age in China and the age range represents the majority of the childbearing population. The final study sample consists of 6,930 women observations.

### Measures

#### Treatment and control group

The CFPS asks respondents to report their pension status, based on the question “What type of pension plan(s) do you have?” in each wave of the survey. According to respondents’ answers, we define the treatment group as those respondents who reported no pension in 2010 but having an NRPS in the wave of 2014. The control group consists of those individuals who were not covered by any pension scheme for the survey in both waves of 2010 and 2014. As a result, the number of cases in the treatment and control group is 4,374 (63.1%) and 2,556 (36.9%), respectively.

#### Fertility outcomes

The first dependent variable for this study, the number of children, measures the number of children among rural married women. In the CFPS data, however, there is no specific question to reflect the number of children born to a woman directly. Alternatively, we create this outcome variable by using the codes from the list of family members to locate the respondent’s each child and to calculate the total number of children the respondent has. We also create an additional outcome variable, having a second child, to investigate the effect of public pension on the likelihood of a second child for rural women. It is measured by a dichotomous variable that equals 1 if the respondent has two or more children and 0 otherwise.

#### Covariates

All regression specifications adjust for several covariates that may confound estimates of the effect of public pension on fertility. Age is a continuous variable that may reflect attitudes toward childbearing at different stages of life. The women’s age at first marriage, a strong correlate of fertility, is also included. To capture the nonlinear effect of education on fertility, the individual education is coded into one of 4-level categorical variable: primary school or less, middle school, high school, and college or more. Since China is a large and diverse country, we control for respondent nationality by a dummy variable, 1 if from an ethnic minority group and 0 for Han nationality. Religion is associated with fertility preferences [22][23] and is measured by another dummy defined from CFPS, 1 if the respondent reports any religious affiliation and 0 otherwise. We capture the effects of health status and health insurance by two measures: a self-rated health status dummy (1 if the respondent reported her health status in “poor” condition) and a health insurance dummy (1 if the respondent was covered by any type of social health insurance). We include employment status, farm work or not working (Yes = 1), self-employed (Yes = 1), wage employed (Yes = 1), important factors shown to affect a woman’s childbearing preferences [24]. Previous studies suggest that saving is strongly correlated to the number of children in the family [16] [25], we control for this effect by including the natural logarithm of household saving per capita. Finally, dummies for the province are included in all models to account for region-specific effects.

### Main empirical strategy

To empirically examine the effect of NRPS on fertility, the average treatment effect on the treated (ATT), a causal impact parameter for comparing the difference between expected fertility outcomes for women with NRPS coverage (the mean of outcomes in the treatment group, i.e. ${\bar{Y}}_{T}$) and the expected fertility outcomes if women had no pension insurance (the mean of outcomes in the control group, i.e. ${\bar{Y}}_{C}$) is estimated. The difference-in-differences (DD) estimation is employed within this study; it is often associated with natural experiments and controls for selection bias on observed factors and unobserved characteristics that are constant over time [26]:

$$ATT_{DD}=({\bar{Y}}_{T,Post}-{\bar{Y}}_{T,Pre})-({\bar{Y}}_{C,Post}-{\bar{Y}}_{C,Pre})$$ (9)

In this setting of policy change, any time-invariant confounders (observed or unobserved) before and after a policy change that might have a potential impact on fertility outcomes can be differenced out by a DD estimator. To augment the robustness of the estimated ATT for NRPS on fertility, a standard DD linear regression model allows for several controls for the differences in observed characteristics between treatment and control group:

$$Y_{it}=\beta_{0}+\beta_{1}(NRPS_{i}\times Post_{t})+\beta_{2}NRPS_{i}+\beta_{3}Post_{t}+\beta_{4}X_{it}+\beta_{5}P_{j}+\mu_{it}$$ (10)

Here, the subscript, *i*, refers to the individual, and *t*, to the period. *Y*_*it*_ is the dependent variable related to the outcomes of female fertility. For the main independent variables, the interaction term, *NRPS*_*i*_×*Post*_*t*_, is the most important regressor and its coefficient, *β*_1_, is the DD estimator that captures the ATT estimator of the NRPS on individual fertility outcomes. *NRPS*_*i*_ is a dummy variable, equaling one for the NRPS treatment group and zero for the non-NRPS control group. *Post*_*t*_ is a dummy variable coded one for the post-treatment period, 2014, and zero for the pre-treatment period in the baseline, 2010. Vector *X*_*it*_ is a group of covariates including individual and family characteristics that may affect the fertility outcomes, and which might have differed between the treatment and comparison groups. *P*_*j*_ is a full set of province dummies accounting for heterogeneity in pension effects by region. Moreover, as the pension program is implemented by county, standard errors in all regressions are adjusted to cluster at the county level.

In a difference-in-differences model, the parallel trend assumption that treatment and control group would not trend differentially during this time period in the absence of a policy change should be satisfied. A common approach to test this assumption is to compare the time trend of fertility outcomes between the treatment and control group before the treatment (i.e., the NRPS in this study) takes place [27][28]. However, we cannot observe such a time trend because the baseline survey of the CFPS was launched in 2010 while the NRPS started in 2009. For this, we will further employ a series of strategies including DD estimation with matching, fixed-effects model, and instrumental variables approach to check the robustness of our main results.

## Results

### Descriptive statistics

Mean of the study variables are presented in Table 1. In the full sample, the average number of children among rural married women is 1.553, and about 55.8% of women report to have a second child or more. For the characteristics of covariates, the mean value of age and the age at first marriage are 34.3 and 21.8 years old, respectively. The proportion of people with middle school or less education is 56.9%, compared with 36.3% and 6.8% of those who achieve a high school or college diploma. The percentage of women reporting to come from an ethnic minority group is 11.6%, and only 3.6% of women claim that they have a religious affiliation. About 10% of respondents reported to be poor health and the participation rate of health insurance in the sample is 90%. The sample contains more people who engage in farm work or not working (63.2%), while the proportions of people working in self-employed (10.6%) and wage employed (26.2%) are much smaller. The annual average household saving per capita is around 3,360 RMB.

**Table 1:** Mean of variables for the full sample and by pension status.

	Full sample	Pre-2010		Diff.(1)	Post-2014		Diff.(2)
		Non-NRPS	NRPS		Non-NRPS	NRPS	
*Fertility outcomes*							
Number of children	1.553	1.207	1.307	***	1.854	1.827	
Before-after difference					0.647	0.520	-0.127
Having a second child	0.558	0.398	0.461	***	0.673	0.679	
Before-after difference					0.275	0.218	-0.057
*Covariates*							
Age	34.38	31.41	32.92	***	35.41	36.92	***
Age at first marriage	21.81	21.78	21.84		21.78	21.84	
Primary school or less (reference)	0.260	0.247	0.268		0.247	0.268	
Middle school	0.309	0.311	0.308		0.311	0.308	
High school	0.363	0.381	0.353	*	0.381	0.353	*
College or more	0.068	0.061	0.072		0.061	0.072	
Minority	0.116	0.135	0.105	***	0.135	0.105	***
Religion (1 = any)	0.036	0.028	0.041	**	0.028	0.041	**
Health status (1 = poor)	0.103	0.071	0.102	***	0.118	0.112	
Health insurance (1 = any)	0.900	0.839	0.893	***	0.857	0.969	***
Farm work or not working (reference)	0.632	0.633	0.660	*	0.608	0.617	
Self-employed	0.106	0.123	0.096	**	0.123	0.096	**
Wage employed	0.262	0.244	0.244		0.269	0.287	
Household saving per capita (RMB)	3,360.7	1,162.3	1,330.9		4,878.6	5,788.1	**
Observations	6,930	1,278	2,187		1,278	2,187	

***, ** and * indicates significance level at 1%, 5% and 10%, respectively.

Table 1 also provides the mean of variables for pre- and post-period by NRPS coverage status, along with the difference of means test between NRPS (treatment) and non-NRPS (control) groups. The results show that, in the pre-period of 2010, women in the NRPS group have more children and are more likely to have a second child than those without NRPS coverage, while there is no significant difference between treatment and control groups in the post-period of 2014. Moreover, compared to the pre-treatment period, the number of children and proportion of having a second child increase in the post-period for both cases of NRPS and non-NRPS, but they increase less in NRPS group (an increase from 1.307 to 1.827, and 0.461 to 0.679) than that of non-NRPS group (an increase from 1.207 to 1.854, and 0.398 to 0.673). These results suggest that a post-treatment effect on women’s fertility outcomes may occur when they had participated in the pension scheme. In the following section, we implement a DD regression to examine the change in outcomes before and after treatment groups by comparing the control group. Additionally, the mean differences between treatment and control group for most covariates are statistically significant and the results change over time for women of NRPS and non-NRPS. We therefore include these controls in all specifications.

### Effect of the NRPS on fertility outcomes

The DD regression results are presented in Table 2. The first two columns display the estimated effects of NRPS on the number of children and the likelihood of having a second child, in the case that covariates are not included. In model (1), the estimated coefficient on NRPS × Post is -0.127 and significantly different from zero, suggesting that the implementation of NRPS has a negative effect on the fertility for the number of children among rural married women. The DD estimation for linear probability regression, in model (2), shows that the NRPS significantly decreases the probability of having a second child by 5.7% for women in treatment groups. These results are consistent with the results from the descriptive statistics, as shown in Table 1, where the ATT could be calculated from the mean differences for fertility outcomes between treatment and control group before and after the policy change. We next re-estimate this effect by including a series of controls and province dummies. As shown in model (3) and (4), the estimated marginal effect of NRPS is significantly 0.119 fewer children and 5.3% decrease in the probability of second child for rural women who had participated in pension program compared to those who had not, which are similar with the results in model (1) and (2). Although the DD estimations indicate that the implementation of NRPS adversely impacts rural women’s fertility, the estimated effect might still be biased because the pension participation is likely to be selected and endogenous with fertility. These findings should be viewed alongside further robustness checks described below.

**Table 2:** DD estimation for the NRPS effect on fertility.

	Number of children (1)	Having a second child (2)	Number of children (3)	Having a second child (4)
NRPS × Post	-0.127***	-0.057***	-0.119***	-0.053***
	(0.036)	(0.016)	(0.037)	(0.016)
NRPS	0.099**	0.063***	0.048	0.015
	(0.047)	(0.023)	(0.036)	(0.019)
Post	0.647***	0.275***	0.496***	0.189***
	(0.035)	(0.015)	(0.035)	(0.015)
Age			0.039***	0.023***
			(0.002)	(0.001)
Age at first marriage			-0.017***	-0.017***
			(0.007)	(0.003)
Middle school			-0.174***	-0.086***
			(0.046)	(0.021)
High school			-0.267***	-0.109***
			(0.049)	(0.024)
College or more			-0.374***	-0.192***
			(0.058)	(0.031)
Minority			0.184*	0.072*
			(0.104)	(0.039)
Religion (1 = any)			-0.032	0.004
			(0.069)	(0.037)
Health status (1 = poor)			-0.052	-0.057***
			(0.034)	(0.018)
Health insurance (1 = any)			-0.046	-0.029
			(0.039)	(0.021)
Self-employed			-0.061*	-0.029
			(0.037)	(0.023)
Wage employed			-0.207***	-0.124***
			(0.030)	(0.016)
Household saving per capita (log)			-0.007***	-0.003**
			(0.003)	(0.002)
Constant	1.207***	0.398***	0.072	-0.327***
	(0.046)	(0.022)	(0.213)	(0.104)
Province dummies	No	No	Yes	Yes
R^2^	0.099	0.060	0.291	0.295
Observations	6,930	6,930	6,930	6,930

***, ** and * indicates significance level at 1%, 5% and 10%, respectively. Robust standard errors with a cluster at the county level are presented in parentheses.

Apart from the pension status, several sociodemographic variables affect fertility as well. We find that older women tend to have more children, but the age at first marriage is negatively associated with the number of children and the probability of a second child to be born. As expected, due to the opportunity cost of childbearing and rearing among educated persons, people with a higher level of education report a lower desire for fertility. Those who come from an ethnic minority group are more likely to have more children, showing a stronger motive for fertility. Women in poor health have fewer children and are less likely to have a second child. However, health insurance has no significant effect on fertility, probably because most of women are insured in the study sample. The fertility-reducing effect varies by employment status. We find a significant reduction in fertility for wage-employed women, but not for the self-employed. Because self-employed jobs are more autonomous and flexible in work arrangements, which make married women have a low opportunity cost during childbearing and they may prefer more children. Finally, the association between household saving and fertility outcomes is found to be significantly negative in both cases of the number of children and the probability of second child, supporting the research findings of Lugauer et al (2019) [16].

### Sample selection

In this section, we address the concern about sample selection that might bias our main results. That is, the estimated effect might be biased if treatment (NRPS) and control (non-NRPS) observations have heterogeneity in initial conditions as treatment is not randomly assigned to women. Following the study of Van den Broeck and Maertens (2015) [29], we combine the DD estimation with propensity score matching (PSMDD) to overcome this problem and check the robustness of the main results. We first estimate the propensity score of NRPS status through a binary logit regression model, including a series of explanatory variables that are both associated with the likelihood of pension participation and fertility outcomes. These explanatory variables are the same as the covariates used in the aforementioned DD regression. Next, using the estimated propensity score we apply a nonparametric Kernel matching approach to derive the matching weights. To check the robustness, we tried different bandwidth (0.06, 0.01, 0.005, 0.001 and 0.0001). As displayed in Table A1 of S1 Appendix, while the sample sizes have various degrees of reduction across different bandwidth, all the point estimates of ATT are similar with only slight changes in significant level in some cases. Since the results appeared robust, we only reported the results of the Kernel matching with a bandwidth of 0.06, a sample of 6,918 observations (4,364 for treatment and 2,554 for the control group) is obtained and used in the DD estimation of pension’s effect on fertility within the common support region.

Results from the PSMDD estimation are still significantly negative but the magnitudes of effects become smaller, as shown in Table 3. Specifically, the estimated marginal effect of NRPS on women’s number of children is -0.08, and the probability of second child for married women is decreased by 4.4% when they are covered by the public pension insurance. We also conduct some analyses for the validity of using propensity matching. Fig A1 of S1 Appendix shows the propensity distribution of the treated and control groups before and after matching. The results demonstrate a noteworthy extension of the common support between the treated and control groups, implying that the overall distributions of the conditional probability to participate in the NRPS are similar between the two groups. Table A2 of S1 Appendix presents the results of covariates balance testing for propensity score matching. The results show that while some variables are significantly different between the unmatched treated and control group, the differences between the two groups for all covariates are no longer significant after matching. This approach resembles a quasi-experimental approach to provide estimates with less selection bias by creating similar characteristics between the treated and control groups.

**Table 3:** Estimated effect of NRPS on fertility from PSMDD.

	PSMDD	
	Number of children	Having a second child
	(1)	(2)
Before diff	0.030	0.011
	(0.049)	(0.025)
After diff	-0.050	-0.033*
	(0.034)	(0.020)
Diff-diff	-0.080**	-0.044**
	(0.036)	(0.017)
Observations	6,918	6,918

***, ** and * indicates significance level at 1%, 5% and 10%, respectively. Robust standard errors with a cluster at the county level are presented in parentheses. A kernel matching approach with bandwidth of 0.06 is employed. Covariates include age, age at first marriage, education, minority, religion, health status, health insurance, employment status, household saving per capita (log), and province dummies.

### Unobserved heterogeneity

One more threat to the main results may come from the potential unobserved heterogeneity, such as unobserved norm and attitude, which may affect both pension participation and fertility preferences. These unobserved factors would cause our estimates of NRPS and fertility effects to be correlated with the error term, resulting in a bias. Following Rokicki et al (2014) and Cheng et al (2016) [30][31], we address this concern by employing the Fixed-effect (FE) regression model:

$$Y_{it}=\beta_{0}+\beta_{1}Pension_{it}+\beta_{2}X_{it}+\beta_{3}W_{t}+c_{i}+\varepsilon_{it}$$ (11)

Where *Pension*_*it*_ is an indicator variable for whether the individual participated in the NRPS, and its coefficient *β*_1_ is our main interest, revealing the effect of NRPS participation on women’s fertility. *X*_*it*_ is the same vector of time-varying covariates from the previous analyses, including age, health status, health insurance, employment status, and household saving per capita (log). *W*_*t*_ is a full set of year dummies, while *c*_*i*_ is the individual fixed effect, which accounts for the unobserved characteristics that are fixed across time.

The first two columns in Table 4 present the results for the FE regression concerning the effects of pension participation on women’s fertility. The NRPS is significantly associated with a 0.102 decrease in the number of children and a 4.7% decrease in the probability of having a second child. It can be seen that, after controlling for individual fixed effect, the magnitudes of effect become smaller than DD estimates. This suggests that part of the fertility-reducing effect of NRPS is indeed driven by the unobserved heterogeneity related to personal pension participation status.

**Table 4:** Estimated effect of NRPS on fertility from FE and 2SLS regression.

	FE regression		2SLS regression	
	Number of children	Having a second child	Number of children	Having a second child
	(1)	(2)	(3)	(4)
Pension	-0.102***	-0.047***	-0.169**	-0.075*
	(0.036)	(0.016)	(0.079)	(0.044)
First-stage coefficients on IV			0.561***	0.561***
			(0.022)	(0.022)
F statistic			444.5	444.5
DWH test			3.69	1.68
Observations	6,930	6,930	3,112	3,112

***, ** and * indicates significance level at 1%, 5% and 10%, respectively. Robust standard errors with a cluster at the county level are presented in parentheses. FE regression includes age, health status, health insurance, employment status, household saving per capita (log), a constant, and controls for individual- and year-specific effects. 2SLS regression include age, age at first marriage, education, minority, religion, health status, health insurance, employment status, household saving per capita (log), province dummies, and a constant.

We also apply the instrumental variables (IV) approach to further reduce the unobserved heterogeneity bias. Several scholars have pointed out that as the NRPS operates at the county level, the implementation status of county pension program has a strong association with the individual participation in NRPS but appears to be the exogenous nature for other individual characteristics that would potentially affect one’s behaviors since the pilot areas of the scheme were decided by central government of China [18][31]. Therefore, we carry out a two-stage least squares (2SLS) procedure using county NRPS status (1 if a county had implemented the NRPS and 0 otherwise) as the instrumental variable for individual pension participation. Because in the study sample there were few counties reporting to be implemented the NRPS at baseline of 2010 and because the pension program was widespread during 2011 and 2014, we only use the survey wave 2014 for 2SLS regression and the sample size contains 3,112 observations. Table A3 of S1 Appendix presents the results for the first stage regression of 2SLS, the estimated coefficient on the instrumental variable is positive and significant (0.561, p<0.01), suggesting that the implementation of county NRPS will increase the likelihood of pension participation for rural women. Moreover, as shown in Table 4, the F statistic (444.5) is much higher than the value of 10 for weak identification and the critical value of 19.93 for 10% IV size [32], indicating that the null hypothesis of a weak instrument should be rejected.

The last two columns in Table 4 show the results for 2SLS regression. NRPS is estimated to reduce rural women’s number of children by 0.169 and significant at the p < .05 level. For whether having a second child, the marginal effect of pension is -0.075 but only marginally significant. A Durbin-Wu-Hausman (DWH) test is used to check the model’s endogeneity, and the null hypothesis that all explanatory variables are exogenous cannot be strongly rejected (1.68 (p = 0.195) and 3.69 (p = 0.054)). The endogeneity problem causes no significant estimation bias in our analysis, and the main results are reliable. Since the major eligibility requirement for NRPS participation is the implementation of the program in that county [31], those living in the non-project counties would have no chance to participate in NRPS. Although the cases that people with NRPS but live in the non-project counties might exist in reality (e.g., immigrants within pension arrangements from project counties), this is not common in China because it is rather inconvenient for people to pay the pension contribution in different places. Moreover, in our sample, we do not find any NRPS participants while their counties have not been included in the pension plan. Therefore, we can be confident that there is few “always-takers” in the sample and the IV estimator should capture the average treatment effect on treated populations, which is more comparable with other estimates.

### Effects by population groups

As the impact of the public pension on fertility is likely to have differential effects in terms of individual characteristics, the sample is stratified by age, education, and household income. Panel A of Table 5 displays estimates stratified by age group, and suggests that the NRPS has a heterogeneous effect on fertility outcomes between younger and older married women. Among those women aged 35 or less, there is a significant decrease, 0.108, in the number of children and a marginally significant decrease of 5.3% in the probability of having a second child. In contrast, there are no significant effects for women aged more than 35 and the marginal effects are smaller. One possible reason is that the fertility behavior, associated with individuals’ age, is more pronounced in the younger people than in the older, so the effect of pension insurance on fertility reduction is larger for younger women than the older.

**Table 5:** Effects by age, education and household income (DD estimation).

*Panel A*: *By age*	Age < = 35		Age > 35	
	Number of children	Having a second child	Number of children	Having a second child
	(1)	(2)	(3)	(4)
NRPS × Post	-0.108**	-0.053**	0.002	-0.009
	(0.050)	(0.026)	(0.052)	(0.023)
NRPS	0.077*	0.013	-0.082	-0.019
	(0.039)	(0.022)	(0.061)	(0.028)
Post	0.629***	0.247***	0.275***	0.107***
	(0.049)	(0.025)	(0.050)	(0.022)
Observations	3,575	3,575	3,355	3,355
*Panel B*: *By education*	Middle school or less		High school or more	
	Number of children	Having a second child	Number of children	Having a second child
	(1)	(2)	(3)	(4)
NRPS × Post	-0.082*	-0.040*	-0.167***	-0.073***
	(0.048)	(0.021)	(0.051)	(0.025)
NRPS	-0.013	-0.008	0.117***	0.040*
	(0.049)	(0.025)	(0.042)	(0.023)
Post	0.405***	0.158***	0.595***	0.218***
	(0.037)	(0.017)	(0.053)	(0.025)
Observations	3,944	3,944	2,986	2,986
*Panel C*: *By income*	Low income		High income	
	Number of children	Having a second child	Number of children	Having a second child
	(1)	(2)	(3)	(4)
NRPS × Post	-0.075	-0.026	-0.130***	-0.063**
	(0.060)	(0.029)	(0.049)	(0.027)
NRPS	-0.001	-0.006	0.089**	0.031
	(0.053)	(0.026)	(0.042)	(0.023)
Post	0.496***	0.184***	0.455***	0.173***
	(0.052)	(0.024)	(0.047)	(0.025)
Observations	3,467	3,467	3,463	3,463

***, ** and * indicates significance level at 1%, 5% and 10%, respectively. Robust standard errors with a cluster at the county level are presented in parentheses. All regression include age, age at first marriage, education, minority, religion, health status, health insurance, employment status, household saving per capita (log), province dummies, and a constant.

The effects of NRPS on fertility vary by different education levels. As shown in the Panel B of Table 5, women with an education level of high school or more have 0.167 fewer children and a 7.3% decrease in the probability of having a second child for the NRPS group than the non-NRPS group. For women with middle school education or less, the coefficients of DD estimation are much smaller and insignificant, implying that the rural women do not have significant responses for either outcome even if they have participated in the pension scheme. The variance may be attributed to the fact that expansion of NRPS benefits in rural areas makes children relatively more expensive among women who have a higher level of education, as they may face a greater opportunity cost of raising children. In this situation, the substitution effect from public pension program is stronger, and thus creates a larger adverse effect on fertility for more educated populations.

Panel C of Table 5 presents the results for women in households with low and high income. We define “low income” as below the mean of per capita household income and “high income” as above the mean. Women who participated in pension insurance from high-income families significantly reduce their number of children by 0.13 and the probability of having a second child by 6.3%. However, NRPS does not significantly affect the fertility outcomes for those in low-income families, with a smaller effect than that of the high-income group. This is likely because the pension participants in high-income families allocate more resources in quality per child instead of child quantity, while those from households with low-income levels might face financial constraints and less likely to change their fertility behaviors.

## Discussion and conclusions

This paper contributes to research on the link between public pension and fertility. We first develop a theoretical model to compare the fertility differences between pension and non-pension scenarios, and then, using Chinese individual-level microdata, we present an empirical analysis for the impacts of NRPS expansion on rural married women’s fertility outcomes. Results from DD estimation show that the implementation of NRPS has a significantly negative effect on women’s demand for the number of children and having a second child. This is in line with the findings of the previous studies [3][5], revealing an inverse correlation between pension and fertility in the cases of advanced countries. We find that, when household saving per capita is controlled for, the effect of NRPS on fertility continues to be negative and significant, suggesting that the fertility-reducing effect is not merely driven by household saving decisions. Moreover, the fertility-reducing effect of NRPS is larger for the younger, more educated women and those in high-income families. The results imply that the need to reach a target population group may be part of what is driving women’s decreased fertility.

Previous research has indicated that the NRPS expansion in rural China significantly improves the health status of children up to 15 years of age, and the health effect is stronger for boys and left-behind children [18]. Combing with our results, this implies that the NRPS leads to family investments in the quality of childcare, which might be an important channel through which the NRPS is associated with reduced fertility because of a tradeoff between child quality and quantity. These results may help us to better understand the role of public pension in escaping poverty in an underdeveloped region. In addition to improving well-being for the elderly, public pension insurance contributes to intergenerational human capital accumulation that may lift families and future generations out of poverty permanently.

Our results in this paper are specific for the case in rural China. The effect of public pension on fertility is likely impinged on by economic conditions and cultural background, which calls for caution in generalizing our results to other countries. One limitation in the present study is that, due to the data limitations, we are not able to identify the parallel trend on the fertility outcomes of the treatment and control group, which is a crucial assumption for DD analyses. If this specific assumption is violated, the estimates of the fertility effects could be biased. Although we can conclude that the public pension is associated with decreased fertility in rural China and several robustness checks, including PSMDD strategy, fixed-effects model, and IV approach, support our findings, these estimates are still subject to some limitations. Additional studies with other data sources and approaches will help to further strengthen our findings.

In spite of the limitations, this study has important implications for social protection in China and other developing countries. First, when evaluating the effect of the pension policy, most previous studies only emphasized the well-being of the elderly while ignored the effect on other family members, for instance, younger women and children. Considerations of such population groups and intergenerational spillovers are needed in the impact evaluations of relevant public pension reform. Second, while implementing a universal coverage pension system is considered to be one major policy to improve social welfare and reduce inequalities, it is worth to pay more attention to the adverse effect of pension on fertility, particularly for the countries facing low fertility problem. In this context, relevant supportive measures are also needed to maintain the number in the labor force and enhance the feasibility and sustainability of public pension systems.

## Supporting information

S1 Appendix (DOCX)
