# Supplementary material for: The fertility effects of public pension: Evidence from the new rural pension scheme in China
Source: PLoS One. 2020 Jun 12;15(6):e0234657. doi: 10.1371/journal.pone.0234657 (PMC7292397; doi:10.1371/journal.pone.0234657)
Supplement: S1 Appendix — (DOCX) [file pone.0234657.s001.docx]

**Appendix**

**Figure A1 Propensity distribution of the treated and control groups before and after matching**

**Table A1. PSMDD estimates using Kernel matching from different bandwidth**

|  | PSMDD | |
| --- | --- | --- |
|  | Number of children  (1) | Having a second child  (2) |
| Bandwidth=0.06 |  |  |
| Diff-diff | -0.080** | -0.044** |
|  | (0.036) | (0.017) |
| Observations | 6,918 | 6,918 |
| Bandwidth=0.01 |  |  |
| Diff-diff | -0.075** | -0.041** |
|  | (0.037) | (0.017) |
| Observations | 6,900 | 6,900 |
| Bandwidth=0.005 |  |  |
| Diff-diff | -0.071* | -0.041** |
|  | (0.037) | (0.017) |
| Observations | 6,880 | 6,880 |
| Bandwidth=0.001 |  |  |
| Diff-diff | -0.061 | -0.048** |
|  | (0.040) | (0.019) |
| Observations | 6,758 | 6,758 |
| Bandwidth=0.0001 |  |  |
| Diff-diff | -0.113** | -0.080*** |
|  | (0.051) | (0.025) |
| Observations | 5,692 | 5,692 |

**Notes:** ***, ** and * indicates significance level at 1%, 5% and 10%, respectively. Robust standard errors with a cluster at the county level are presented in parentheses. Covariates include age, age at first marriage, education, minority, religion, health status, health insurance, employment status, household saving per capita (log), and province dummies.

**Table A2. Covariates balance testing for propensity score matching**

| Variable | Unmatched | Mean | | % bias | % reduct  \|bias\| | *t*-test | |
| --- | --- | --- | --- | --- | --- | --- | --- |
|  | Matched | Treated | Control |  |  | *t*-statistics | *p*>\|t\| |
| Age | U | 36.925 | 35.408 | 24.600 |  | 7.040 | 0.000 |
|  | M | 36.921 | 37.015 | -1.500 | 93.8 | -0.510 | 0.608 |
| Age at first marriage | U | 21.839 | 21.785 | 2.500 |  | 0.700 | 0.484 |
|  | M | 21.838 | 21.823 | 0.700 | 72.9 | 0.220 | 0.826 |
| Middle school | U | 0.308 | 0.311 | -0.600 |  | -0.180 | 0.858 |
|  | M | 0.308 | 0.310 | -0.400 | 41.7 | -0.120 | 0.903 |
| High school | U | 0.353 | 0.381 | -5.900 |  | -1.690 | 0.092 |
|  | M | 0.353 | 0.358 | -1.100 | 81.9 | -0.360 | 0.721 |
| College or more | U | 0.072 | 0.061 | 4.300 |  | 1.210 | 0.225 |
|  | M | 0.071 | 0.069 | 0.800 | 81.5 | 0.260 | 0.797 |
| Minority | U | 0.105 | 0.135 | -9.300 |  | -2.680 | 0.007 |
|  | M | 0.105 | 0.107 | -0.500 | 95.1 | -0.160 | 0.873 |
| Religion (1 = any) | U | 0.041 | 0.028 | 7.100 |  | 1.970 | 0.049 |
|  | M | 0.041 | 0.034 | 3.700 | 47.8 | 1.180 | 0.238 |
| Health status (1 = poor) | U | 0.112 | 0.118 | -1.900 |  | -0.550 | 0.584 |
|  | M | 0.112 | 0.114 | -0.700 | 63.8 | -0.230 | 0.817 |
| Health insurance (1 = any) | U | 0.969 | 0.857 | 40.500 |  | 12.560 | 0.000 |
|  | M | 0.969 | 0.969 | -0.100 | 99.8 | -0.040 | 0.965 |
| Self-employed | U | 0.096 | 0.123 | -8.400 |  | -2.430 | 0.015 |
|  | M | 0.097 | 0.095 | 0.500 | 94.0 | 0.180 | 0.858 |
| Wage employed | U | 0.287 | 0.269 | 3.900 |  | 1.110 | 0.268 |
|  | M | 0.287 | 0.287 | 0.000 | 99.5 | 0.010 | 0.994 |
| Household saving per capita (log) | U | 3.968 | 3.663 | 7.000 |  | 1.980 | 0.048 |
|  | M | 3.965 | 3.912 | 1.200 | 82.6 | 0.400 | 0.689 |
| Sample | Ps R2 | LR chi2 | p>chi2 | MeanBias | MedBias | B | R |
| Unmatched | 0.046 | 210.280 | 0.000 | 9.700 | 6.500 | 49.6 | 0.37 |
| Matched | 0.000 | 2.630 | 0.998 | 0.900 | 0.700 | 4.9 | 1.06 |

*Notes:* B represents absolute standard deviation and R denotes the standard deviation ratio.

**Table A3. 2SLS estimation first-stage regression results on the likelihood of NRPS participation**

|  | NRPS participation |
| --- | --- |
| Age | 0.008*** |
|  | (0.002) |
| Age at first marriage | -0.001 |
|  | (0.004) |
| Middle school | 0.019 |
|  | (0.022) |
| High school | 0.031 |
|  | (0.026) |
| College or more | 0.024 |
|  | (0.041) |
| Minority | 0.026 |
|  | (0.042) |
| Religion (1 = any) | 0.036 |
|  | (0.045) |
| Health status (1 = poor) | -0.022 |
|  | (0.027) |
| Health insurance (1 = any) | 0.296*** |
|  | (0.027) |
| Self-employed | -0.006 |
|  | (0.032) |
| Wage employed | 0.031 |
|  | (0.020) |
| Household saving per capita (log) | 0.003 |
|  | (0.002) |
| Implementation status of NRPS in county (1=yes) | 0.561*** |
|  | (0.022) |
| Constant | -0.219** |
|  | (0.108) |
| Province dummies | Yes |
| R^2^ | 0.251 |
| Observations | 3,112 |

**Notes:** ***, ** and * indicates significance level at 1%, 5% and 10%, respectively. Robust standard errors with a cluster at the county level are presented in parentheses.
